# Recent HIV Infection: Diagnosis and Public Health Implications

**DOI:** 10.3390/diagnostics12112657

**Published:** 2022-11-01

**Authors:** Georgios K. Nikolopoulos, Andreas G. Tsantes

**Affiliations:** 1 Medical School, University of Cyprus, Nicosia 2029, Cyprus; 2 Microbiology Department, “Saint Savvas” Oncology Hospital, 11522 Athens, Greece

**Keywords:** HIV, recent infection, HIV incidence, prevention

## Abstract

The early period of infection with human immunodeficiency virus (HIV) has been associated with higher infectiousness and, consequently, with more transmission events. Over the last 30 years, assays have been developed that can detect viral and immune biomarkers during the first months of HIV infection. Some of them depend on the functional properties of antibodies including their changing titers or the increasing strength of binding with antigens over time. There have been efforts to estimate HIV incidence using antibody-based assays that detect recent HIV infection along with other laboratory and clinical information. Moreover, some interventions are based on the identification of people who were recently infected by HIV. This review summarizes the evolution of efforts to develop assays for the detection of recent HIV infection and to use these assays for the cross-sectional estimation of HIV incidence or for prevention purposes.

## 1. Introduction

Human Immunodeficiency Virus (HIV) infection remains a major public health issue globally. According to recent estimates from the joint United Nations program on HIV/AIDS (UNAIDS), approximately 38 million people were living with HIV in 2020, while more than half a million people died that year because of complications related to the Acquired Immune Deficiency Syndrome (AIDS) [1]. Nevertheless, major strides have been made on multiple fronts, and HIV infection has become a manageable chronic condition, especially due to the advent of very potent antiretroviral drugs [2]. As a matter of fact, despite disparities in survival rates across population groups and geographical settings, the expected life span of an HIV-infected person has increased remarkably following the introduction of antiretroviral treatment (ART), being almost equal to that of a non-infected person of the same age [3,4,5]. 

Apart from condom use and behavioral interventions that have proven prevention capacity [6], there are also many ART-based interventions that have shown high rates of effectiveness at reducing the likelihood of HIV transmission including Pre-Exposure Prophylaxis (PrEP) [7,8,9,10] and Treatment as HIV Prevention (TasP) [11,12,13,14,15]. However, challenges remain because HIV incidence globally still exceeds 1.5 million new infections [1], with explosive outbreaks on some occasions often created or facilitated by economic, social, and political crises [16,17,18]. Moreover, the efforts of the scientific community to develop effective vaccines have, to a large extent, been disappointing [19]. Currently, as of fall 2022, only one phase 3 clinical trial on a promising HIV vaccine is ongoing and the results are expected in 2024 (https://www.avac.org/trial/hpx-3002-hvtn-706-mosaico, accessed on 17 September 2022). 

Consequently, further research and efforts are needed in order to accomplish the goal of putting an end to AIDS as a public health threat by 2030 [20,21]. Of importance, the diagnosis of HIV infection and the start of ART soon after the transmission event comprise an essential pillar of the strategy to end AIDS [21].

## 2. What Is Recent HIV Infection and Why It Is Important

An HIV infection is considered chronologically recent when it occurred within the last six to twelve months [22,23,24]. This recent period is characterized biologically by initially high levels of HIV replication and concentration of HIV markers in biological sites and fluids followed by the gradual development of the adaptive immune response [25,26,27,28,29]. It includes acute HIV infection, which refers to the first weeks (2-4 weeks) after HIV acquisition before the appearance of antibodies [28,29,30,31,32]. During the recent phase, antibodies, when they emerge, have not yet reached their full developmental and effectiveness potential, as their production and maturation process evolves gradually over the course of HIV infection [32,33]. Antibody levels and avidity tend to stabilize approximately one year after infection. The breadth of antibody reactivity (i.e., the number of unique HIV epitopes that are targeted) also increases early in the infection but reaches a set point, remaining stable or decreasing thereafter [34]. By the time the antibody response is effective, it is too late to prevent the establishment of the infection [27,29,35,36]. 

The identification of recent HIV infection is important for two main public health reasons: (i) It helps detect people during a period of increased transmissibility, which can guide intervention efforts making better use of scarce resources, and (ii) it allows real-time tracking of trends in new infections at the population level, which helps with the timely evaluation of prevention efforts [11]. 

Some newly HIV-infected people, especially if they remain undiagnosed, are likely to continue risky behavioral practices for a period after their infection that de facto led to their infection or the infection of their sexual or injecting partners [30,37,38]. The recent period of HIV infection is likely to interact with the concurrency of sexual partnerships and the high rates of partner change, amplifying their effect on onward transmissions [39,40]. Beyond risky behavioral patterns, intense HIV replication and high concentration in biological fluids likely comprise the primary cause of the higher infectiousness of the acute/recent period of HIV infection [25,30]. Analyses of cervical secretions of African women showed higher viral load in the acute phase than subsequently [41]. Similarly, viral concentration in the semen and blood of African males with acute HIV infection was higher than that among those with chronic infection [42]. An early epidemiological study using couples whose partners had different infection statuses provided evidence that HIV viremia was a strong predictor of HIV transmission [43]. Another study, also in serodiscordant heterosexual couples, found that the early and late stages of HIV infection were the most infectious [44], although subsequent analyses argued that the infectivity of the acute phase in that study had perhaps been overestimated [45]. Other methods of analysis corroborated the role of recent HIV infection. Phylogenetic research in HIV-infected men who have sex with men (MSM) in the United Kingdom showed that transmitters were more likely to be in the recent period of their infection [22]. Likewise, other phylogenetic analyses found that nearly half of onward transmission events could be from recently HIV-infected people [46,47]. Modelling work has produced interesting but variable findings in this field. An early mathematical model for MSM attributed approximately one-third of transmissions among causal partners to the recent phase of HIV infection, but the contribution of recently infected people to infections that occurred among steady partners was estimated to be considerably lower [48]. Mathematical models run for Malawi, a generalized HIV epidemic setting, attributed almost 40% of infections to sexual contact with recently HIV-infected people [49]. Another modelling study for South Africa, however, raised concerns over the contribution of the early HIV infection period to long-term intervention (TasP) effects [50,51]. Modelling work for people who inject drugs (PWID) argued that the first months of HIV infection are more relevant in outbreaks and likely less important in mature epidemics, where a much lower proportion of all transmissions was attributed to an index case being in his/her early period of HIV infection [52]. In summary, despite differences in the relative contribution of the acute and recent stages of HIV infection, a considerable amount of evidence supports the increased infectiousness of people experiencing acute/recent HIV infection. 

Traditional case-based surveillance often lacks the ability to capture recent transmission phenomena as it suffers from reporting delays or even fails to include the undiagnosed fraction of the HIV-infected population [53]. AIDS cases reflect infections that happened long ago as survival has increased remarkably in the era of ART, while HIV reporting, despite being more useful than AIDS notification alone, is seriously impacted by patterns of testing and reporting [53,54]. HIV prevalence studies are extremely useful in measuring the burden of HIV infection in at-risk sub-populations, including those who have not yet been diagnosed, but they capture a mixture of both incident and prevalent cases. Moreover, HIV prevalence is likely to increase due to the ART-based improved survival even when incidence plummets. Therefore, the detection and measurement of recent infections can serve HIV surveillance by providing a more accurate picture of new transmission events [54]. Beyond assisting in incidence measurement and serving surveillance purposes, testing for recency could also help public health personnel identify clusters and geographical hotspots of recent HIV transmission, and conduct contact tracing [55,56]. All these pieces of information could be extremely useful for designing and evaluating prevention interventions [31].

## 3. Identification and Diagnosis of Acute/Recent HIV Infection

**Figure 1:**
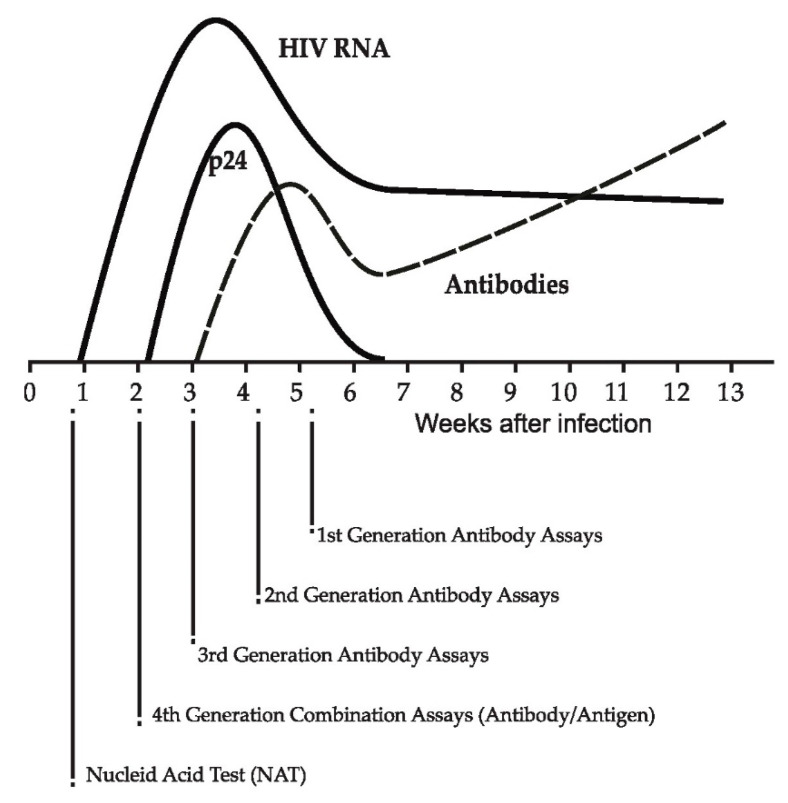
Viral and serological markers during recent HIV infection and evolution of HIV diagnostic assays (adapted from [57]).

Symptoms during recent HIV infection are lacking or are not specific enough to attract clinical suspicion and facilitate diagnosis. There are, however, several serological and non-serological biomarkers that indicate recent HIV infection including HIV RNA, HIV protein 24 (p24), and antibodies [25,31,32] (Figure 1). Assays have been created to detect and/or measure these biomarkers (Table 1) [31]. 

HIV has two subtypes: HIV-1 and HIV-2. HIV-1 is the dominant subtype globally and is further classified into groups M, N, O, and P. Infection by HIV subtype 2 is endemic in West Africa and characterized by slower progression to advanced disease [71,72]. HIV-2 infection is suspected in people of West African origin, those who have had sexual contact with or who have shared injection equipment with people of West African origin, and people who live in countries with strong socioeconomic connections with West Africa (for example, Spain, Portugal, France).

The presence in plasma/serum of viral markers such as HIV RNA or p24, without the detection of antibodies, is an indication of acute infection (pre-seroconversion period) [22,28]. However, the measurement of HIV RNA (nucleic acid amplification test—NAT) is expensive while the presence of free p24 is transient, since it rapidly binds to developing antibodies forming immunocomplexes and cannot be detected unless the relevant assay is able to disrupt the bond [28]. Beyond antibody-antigen disruption, the failure of tests to detect p24 has also been associated with HIV subtypes, use in low-prevalence or resource-poor settings, and stability of test components and targets [32]. Testing for HIV RNA in pooled samples negative for antibodies has been used as a method to improve the detection of acute infection and decrease costs [28,29].

Modern diagnostic strategies aim to maximize the efficiency and accuracy of the diagnosis of acute HIV infection. They involve an initial combination fourth-generation assay that simultaneously detects p24 and antibodies [28,57,73]. If the combination assay is reactive, an antibody differentiation test is used. In case the differentiation assay does not react to HIV-1 or HIV-2 antibodies or the result is indeterminate, NAT follows, which, if positive, indicates acute HIV infection [28,57]. If recent exposure to HIV is suspected or conducted, NAT is used even when the initial combination assay is non-reactive. HIV screening using a combination assay following a rapid negative test in a high-prevalence setting showed promising results detecting more than 80% of acute infections that were identified by pooled NAT [30]. Fifth-generation immunoassays have also been developed, which are able to distinguish p24 and antibodies to HIV-1 or HIV-2 in a single test without needing an antibody differentiation assay [31,57,74].

The new diagnostic algorithm, mentioned above, considers HIV-2 infection. The Geenius HIV 1/2 Supplemental Assay, which uses a closed lateral flow cartridge with a dual-path platform to detect antibodies against recombinant or synthetic peptides for HIV-1 and HIV-2, has received approval to differentiate HIV-1 infection from HIV-2 infection [31]. When the HIV-2 test on the HIV-1/HIV-2 differentiation immunoassay gives an indeterminate result, HIV-2 NAT (information here: https://www.wadsworth.org/programs/id/bloodborne-viruses/clinical-testing/hiv-2-nucleic-acid, accessed on 17 September 2022) is recommended because HIV-1 NAT does not reliably detect or quantify HIV-2 RNA [75]. It is important to mention, however, that more than one-third of people who are HIV-2 infected and ART-naïve have undetectable HIV-2 RNA levels [75,76]. Therefore, a negative HIV-2 NAT does not rule out HIV-2 infection [75].

A salient characteristic of recent HIV infection (post-seroconversion period) is the immaturity of the evolving antibody response [33]. This initial immaturity includes lower levels/titers of IgG antibodies, reduced avidity of antibodies with antigens, and fewer IgG antibodies specifically against HIV compared to the overall IgG amount [31]. Specific assays take advantage of the quantitative and qualitative characteristics of the evolution of the antibody response and are able to detect recent infections in the post-seroconversion period [55,77]. The first effort of this kind was based on using sensitive/less sensitive (detuned) enzyme immunoassays (EIA) on specimens that had been collected cross-sectionally [58]. The concept of the method was that antibody titers are expected to be lower during recent HIV infection. Therefore, using this method, an infection was considered recent if the specimen was reactive on the sensitive EIA but the result became non-reactive when a modified, less sensitive version of that EIA was used. This method showed variability within runs and laboratories, was cumbersome requiring multi-step dilution, and did not perform well with HIV subtypes other than B [31,59,63]. Similar assays were developed later but their performance also varied considerably across HIV subtypes and have not thus been widely used [60,61].

The IgG-capture BED-EIA was another recency test that was based on the produced number of IgG antibodies directed against the HIV glycoprotein 41 (gp41), as a proportion of total IgG, with that proportion increasing over time following seroconversion [31,63,78]. To overcome a suboptimal subtype-dependent performance, BED uses a trimeric branched peptide from the immunodominant region of the gp41 of HIV-1 subtype B, a Circulated Recombinant Form (CRF_01 AE), and subtype D [33]. The three peptides gave the name BED to the assay. BED-EIA classifies an HIV infection as recent when the ratio of HIV-specific IgG to total IgG in the blood is low, which corresponds to a normalized optical density (ODn) below a pre-set threshold (0.8) [79]. This approach has been used widely but suffers from limitations including performance variability across populations and therefore needs adjustments when applied for incidence estimations [80,81,82,83,84,85,86,87,88,89,90,91].

Avidity assays comprise a rather reliable approach in the field of laboratory detection of recent HIV infection that is based on a functional property of antibodies, i.e., the strength of their binding with antigens. Early in HIV infection, the bond is not strong enough, which allows the easier dissociation of antigen–antibody complexes [31]. As antibodies mature, they become more resistant to disruption treatment and the avidity index (AI) increases. The AI is calculated in two-well assays as a ratio of OD values from one well treated with a reagent to dissociate low-avidity antibodies and a control well that remains without treatment. A commercial assay of this kind is the BioRad avidity test [79]. It is a modified version of an HIV-1/HIV-2 EIA and involves the testing of samples with and without diethylamine. The AI of that assay is calculated by dividing the reactivity of the treated over the untreated aliquot with values below 40% indicating a recently acquired HIV infection. In a similar concept, Architect avidity is a modified version of a combination assay where each specimen is tested in the presence and absence of guanidine [92]. The AI of that assay is the reactivity ratio of treated to untreated aliquots with values below 80% indicating recent HIV infection. Another assay that has been used in practice and calculates AI, as a ratio of signal-to-cutoff (S/C) values of the sample incubated in guanidine to the S/C of the sample incubated in phosphate-buffered saline, is Vitros Avidity [93]. Generally, assays that calculate AI have limitations including the need for automated systems and two wells, with the latter contributing to heightened variability [31]. There is also the immunochromatographic assay Geenius HIV 1/2 Supplemental Assay that measures the intensity of bands specific for antibodies to HIV-1 and HIV-2 antigens and for total IgGs to a control protein. A Geenius Index (GI) has been developed that is defined as the sum of the intensities of gp41, gp160, and p31 bands divided by the intensity of the control band, with a value below 1.5 interpreted as indicating recent HIV infection [94].

Limiting Antigen (LAg) avidity assay (LAg-EIA) is the primary representative of the avidity-based approach to distinguishing recent and long-standing HIV infections. Although a two-well system had also been developed, the currently available commercial assay is a single-well approach with equally good, if not better, performance [64]. The well of LAg-EIA is coated with a limiting amount of antigen that is expected to permit the binding of only highly avid antibodies, while a low-PH buffer is also used to further dissociate weaker, low-avidity antibodies. LAg-EIA employs a chimeric recombinant gp41 that covers multiple subtypes and CRFs of HIV-1 group M [31,64,65]. A LAg-EIA ODn of less than 1.5 represents recent HIV infection [66,79]. Despite its strengths and dominance in the field, LAg-EIA was less than optimal when used alone in assessing and distinguishing recent and long-term infections, and in incidence estimations [79,82,95].

Point-of-care (POC) testing offers an easy-to-perform and less expensive diagnostic capacity at the community level and, in general, in settings where centralized reference laboratory facilities are usually absent. Rapid tests for recent infection could be useful for surveillance and intervention purposes with real-time identification of HIV transmission events and the associated risk factors, and fast implementation and evaluation of targeted prevention programs including contact tracing [96]. A rapid test to simultaneously confirm HIV diagnosis and identify recent infection that is based on the method of limiting antigen has already been developed [31,97]. It is a strip-test that includes an additional line with the antigen at a limiting concentration to differentiate recent from long-standing HIV infection. The test uses the same peptide of the LAg-EIA but in a rapid test format and has both visual and reader-based interpretation [97,98]. According to the visual reading, the presence of all three lines (control line (CL), positive verification line (PVL), and long-term line (LTL)) indicate long-term HIV infection; the presence of the two lines CL and PVL indicate recent HIV infection; and the presence of only CL supports the absence of HIV infection. The strip reader measures the intensity of the three lines (in intensity units-IU). A recent analysis of specimens representing multiple subtypes and diverse geographic origins showed that the sensitivity and specificity of that rapid recency test for HIV diagnosis were higher than 99% and 98.5%, respectively, an overall agreement with LAg-EIA higher than 91%, and a high-level concordance between visual and strip reader-based results [31,98].

In general, the performance of serological assays for detecting recent HIV infection in the post-seroconversion period depends on the HIV infection stage, receipt of antiretrovirals as treatment or prevention, variability associated with HIV subtypes, and individual variability in immune response [33,55,79]. In particular, the wrong classification of long-term infections as recent increases in HIV-infected people with low CD4 T-cell counts or in HIV-infected people on antiretrovirals, including those who started ART soon after their infection [79,88,89,93,99,100,101]. Increased rates of misclassification have also been observed in elite controllers who naturally suppress HIV viral load [79,102]. The misclassification of longstanding infections as recent seems also to be increased in people with subtype D HIV infection, which could be attributed to the weaker antibody response at the beginning of infection with subtype D that remains over its course [103,104,105,106].

## 4. Use of Recency Assays in HIV Incidence Estimations

Assays developed to detect a marker of recent HIV infection have been used to estimate HIV incidence [31,55]. This practice is being increasingly promoted within conventional surveillance systems [54,107]. Traditionally, in epidemiology, cohort studies comprise the primary research design to estimate incidence [108,109]. A group of HIV-free individuals is followed over time and tested periodically to detect seroconversions. Incidence is easily calculated by dividing the number of new infections by the total person-time of observation. Adjustments could be made regarding the hypothetical time of infection often assumed to have occurred in the middle between two successive HIV tests (negative result followed by a positive result). However, prospective cohort studies are demanding in terms of logistics, and they are also expensive and time consuming [31,54]. Alternatively, HIV incidence can be calculated indirectly if age-specific and/or repeated cross-sectional estimates of HIV prevalence are known [110,111,112,113,114]. This approach, however, still requires substantial and sometimes longitudinal epidemiological information across multiple age groups and may give poor proxies of recent incidence [105]. Prevalence, mortality, ART data, AIDS and/or HIV cases, and CD4 T-cell counts could also feed mathematical models in order to produce incidence estimates [54,114,115,116,117]. Estimates of mathematical modelling, however, could be potentially biased if incorrect model assumptions are made or unreliable input data are used, while the methodology is too complex for the average public health practitioner to comprehend. Moreover, the retrospective modelling estimations of incidence are perhaps not available on time, and this delay is not helpful when immediate interventions along with their evaluations are needed.

In this context, laboratory assays based on antibody detection in cross-sectional specimens remain attractive, offering an alternative, less expensive, and conceptually simple method of estimating incidence [118]. Of course, testing for viral markers could also be of use for incidence estimates using cross-sectional designs [119]. However, the pre-seroconversion period is short, introducing significant variability in the estimation of the mean period of acute infection and thus impacting the precision of the incidence estimates. Furthermore, even areas with very high HIV prevalence would require HIV RNA or p24-based testing of a vast amount of specimens to achieve the appropriate confidence in the incidence estimates [31,91]. This creates logistical and practical issues, while the cost increases considerably.

The calculation of incidence from cross-sectional designs using assays to detect recent HIV infection considers two important parameters that are closely related to the sensitivity and specificity of a classification procedure: The mean duration of recent infection (MDRI) and the False Recent Ratio (FRR). MDRI is the average time a person stays in a recent state within time T after he/she got infected (2 years is recommended for T) [106,120]. Ideally, for statistical robustness, the MDRI should be at least six months in duration with low context-related variability. It seems, however, that it does vary across different contexts with divergent subtypes [66,121]. The calculation of MDRI needs repeated measurements of recency biomarkers in panels of HIV seroconverters and specific statistical methods including linear and non-linear mixed models and techniques of survival analysis [105,120,122,123]. FRR is the proportion of people who are not recent in reality but the result of a relevant assay indicated recent HIV infection (i.e., similar to 1 minus specificity in individual diagnostics) [120]. Ideally, it should be calculated in a representative sample of people whose duration of HIV infection is longer than T, the selected time boundary of recency.

Incidence can be calculated using the following simple estimator (snapshot): I = R / (μ × N), where R is the number of HIV-positive and recently infected people (based on a recency test), N represents HIV-negative people, and μ is the average duration of infection among recently infected people [124,125]. It is suggested that the snapshot estimator is unbiased when the incidence does not change over the period of time that precedes a cross-sectionally collected sample. Moreover, it assumes that the duration-specific test-recent probability function goes to zero, indicating zero test-recent probability for a subject infected long enough [125]. On most occasions, however, FRR is not 0, showing variability across demographic and epidemiological contexts [23]. Therefore, relying on a cross-sectional sample of people who were tested for HIV and subsequently for recent infection, if found HIV-positive, the average incidence (I) over a pre-specified time T following the infection with HIV can be calculated using a generally adjusted estimator, as follows: I = (R – ε × P)/[(Ω_Τ_ – ε × Τ) × Ν] where R is the number of HIV infections classified as recent, ε is the probability that the recency test classifies as recently HIV infected a person with a duration of HIV infection longer than T (i.e., FRR), Ω_Τ_ is the MDRI, and P and N are the numbers of HIV-positive and HIV-negative participants in the sample [105,122,126]. For the adjusted estimator, the assumption is that the duration-specific test-recent probability function is constant in the tail, which allows for a non-zero test-recent probability of a long-term infected subject. In other words, the false-recent test probability is not associated with the duration of the infection. The assumptions and statistical properties of the two above-mentioned estimators have recently been described in detail [125]; when the assumptions on the constant incidence and recency test characteristics do not hold, the adjusted estimator is more robust than the snapshot estimator.

**Figure 2:**
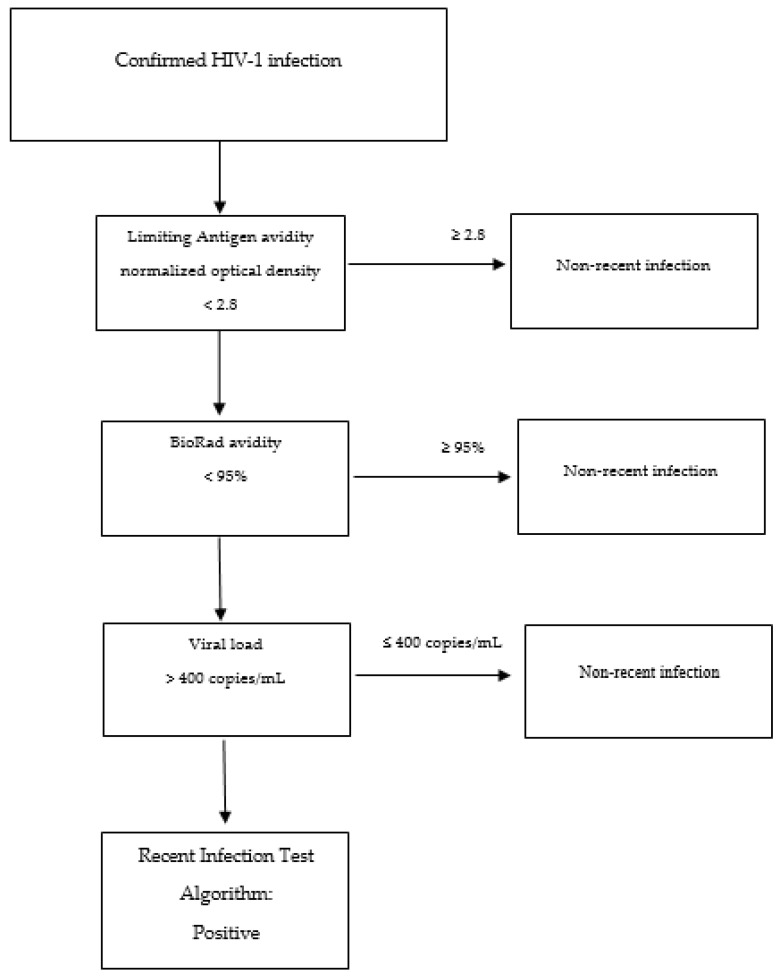
Example of the flow of a recent infection test algorithm.

Stand-alone assays fail to achieve simultaneously sufficient MDRI (>6 months) and low FRR in all contexts (ideally 0 but certainly less than 2%) [106]. Therefore, combinations of serological and non-serological assays and other information have been used to improve the performance of incidence estimations (Recent Infection Test Algorithm—RITA) (Figure 2) [127,128].

Testing algorithms often include information on viral load in particular but also on ART and have given quite reliable results in many settings [83,91,92,95,106,129,130,131] (Table 2). Of interest, in a very large household-based study of more than 18,000 individuals in a high-prevalence setting (Swaziland), a RITA based on Lag (≤1.5 ODn) and viral load (≥1000 copies/mL), along with NAT testing of HIV-negative people for acute infection, produced very similar estimates of incidence (2.6%) compared to that based on the follow-up observation and testing of the same individuals (2.4%) [108,132]. More recent work has studied multiple-assay algorithms (MAAs), with various combinations of a greater number of assays, for population-level cross-sectional estimations of HIV incidence. For instance, an MMA that involved LAg-Avidity < 2.8 ODn, BioRad-Avidity < 95%, and viral load > 400 copies/mL performed well, providing a precise and accurate estimate of HIV incidence in a setting where HIV subtype C was prevalent [128]. The statistical analysis of that work included the estimation of the proportion of samples classified as MMA-positive as a function of time after seroconversion (φ(t)). This function φ(t) was used to estimate the mean window period (the average time someone was classified as MAA-positive) and the shadow, which tells us how far back in time incidence is measured. Although the MDRI and mean window period are both estimated as areas under the probability curve, they may differ since the MDRI curve is usually truncated at 2 years after seroconversion [128]. Recent research has also presented a new incidence estimator for RITA in the context of cross-sectional surveys that depends only on reference FRR and MDRI, i.e., external parameters calculated in a population of undiagnosed, non-elite controllers, and without receiving treatment or having developed AIDS [133].

## 5. Interventions Based on Assays to Detect Recent HIV Infection

Beyond applications for the cross-sectional estimation of HIV incidence, the knowledge of recent acquisition of HIV helps detect and understand hot spots of viral transmission and implement prevention programs. The number of this type of analysis and intervention has been increasing. For example, targeting a cohort of MSM in Thailand who had recently acquired HIV and getting them linked to care resulted in huge reductions in their viral load and, consequently, decreases in the estimated number of transmissions in the first year of their infection from 27.3 without intervention to 5.9 [134]. Another group in China used various testing algorithms based on biomarkers of early infection and found that recent HIV transmission was significantly associated with MSM and a young group of 15 to 24 years old [56]. In an Indian setting, researchers implemented a LAg-EIA-based RITA and found a high prevalence of recent HIV infection among female sex workers, MSM, and PWID [135]. Researchers in Kenya tested more than 1000 HIV-positive specimens for recent infection and found that living in certain areas including the capital city of Nairobi, being currently married or widowed, having more than two sexual partners in the last year, having condomless sex, coinfection with another sexually transmitted pathogen, and being younger than 30 years old with a lack of male circumcision were associated with recent infection [136]. The Transmission Reduction Intervention Project was a remarkable, multi-site effort (Athens, Greece; Odessa, Ukraine; Chicago, United States) to prevent HIV transmission by focusing on recently HIV-infected people [137,138,139,140]. The project staff used LAg-EIA and other clinical and laboratory information to identify recent HIV infections among PWID and contacted network-based contact tracing [129,141]. This network-based intervention focusing on recent HIV infection was successful in identifying more people who had acquired HIV recently, linking them to care, and reducing their viral load to almost undetectable levels [142]. Without any evidence of serious stigmatizing phenomena or other adverse events, the yield of new recent and undiagnosed HIV infections was higher in the networks of recently HIV-infected participants who were used as seeds in the intervention than in the networks of a control group consisting of participants with long-term HIV infection who also served as seeds for the network-based contact tracing [141,143]. The efforts to identify and intervene timely with recently HIV-infected people would perhaps further be facilitated if recency assays were to assure appropriate assessments at the individual level, thus allowing the provision of diagnostic information to a single person. In this respect, many research groups try to improve the properties of MAAs. For instance, a recent study used, in different combinations, two avidity assays (LAg-EIA and BioRad avidity), a LAg version for rapid recency testing, a multi-drug assay to identify uptake of ART among seroconvertors, and viral load in an attempt to identify an MAA for optimal individual-level assessment [127]. All MAAs in that study showed low FRRs (0.2–1.3%) but the True Recent Ratio (TRR or sensitivity in individual-level diagnostics) was less than 50%.

## 6. Conclusions

It has been now more than 25 years since the first publications related to HIV incidence estimation using an assay that was based on the early period of HIV infection [58,144]. Since then, several innovative recency assays have been developed, which have, however, exhibited suboptimal performance when used alone. Other diverse approaches are also being investigated, including circulating cellular microRNAs in plasma as biomarkers of HIV infection, the detection of recent HIV infection based on naturally inspired synthetic oligomers, antibody reactivity to a panel of different HIV peptides that can better predict the duration of HIV infection, studying the genetic diversity of HIV during the early period of infection, which is expected to be less than that in later stages, POC rapid testing of HIV-1 RNA or DNA using new technologies, the use of gold nanocluster immunoassays, etc. [31,34,67,68,69,70,145,146,147,148,149,150]. The World Health Organization (WHO) long ago set up a technical group on HIV incidence assays. The Consortium for the Evaluation and Performance of HIV Incidence Assays (CEPHIA) was also established, which, among others, regularly validates and evaluates the characteristics of existing assays [23,24,147]. Therefore, profound knowledge and experience have been accumulated. The above-mentioned scientific groups should continue their work and receive the appropriate support and funding. In terms of incidence calculations, estimators are available, which need to consider MDRI and FRR specific to the epidemic context, and a couple of MMAs seem to work satisfactorily in a variety of settings. There is also a strong move towards incorporating the monitoring of recent HIV infections in routine surveillance and using recency assays for the identification of transmission hotspots and for prevention interventions. The great challenge, however, remains the development of an MAA with optimal characteristics for the diagnosis of recent HIV infection on an individual patient basis. This challenge is gaining momentum as it will allow physicians and public health practitioners to formally inform recently HIV-infected people of their status and consequently enhance partner notification and counseling in order to help these people make behavior changes and break networks of HIV transmission.

## Figures and Tables

**Table 1:** Assays that have been used for the detection of recent HIV infection.

Assay	Period	Biomarker	Example-Technique
Antigen-based test	Pre-seroconversion (0–4 weeks)	Viral protein (p24)	Fourth generation antibody/antigen combination assay [28]
Nucleic acid amplification test (NAT)	Pre-seroconversion (0–4 weeks)	Viral genome	HIV RNA PCR test [28]
Detuned:	Post-seroconversion	Host marker—rising antibody titer after seroconversion	Sensitive/less sensitive EIA (first generation EIA) [58,59,60,61,62]
BED-EIA:	Post-seroconversion	Host marker—Relative ratio of HIV specific IgG to total IgG	Capture enzyme immunoassay using a multi-peptide from subtypes B and D, and Circulating Recombinant Form_01 AE [62,63]
Avidity EIA:	Post-seroconversion	Host marker—Avidity of antibodies with antigens	Limiting Antigen Avidity assay (LAg-EIA) [64,65,66]
Other methods/new technologies	- Based on the genetic diversity of HIV (people with recent HIV infection probably have less genetic diversity than people with long-term HIV infection) [67,68,69] - Based on “HIV serosignature” (measuring antibody reactivity to a panel of peptides associated with recent HIV infection) [34] - Gold nanocluster immunoassay (gold nanoclusters conjugated with streptavidin are used as ultrasensitive fluorescent sensors for the detection of HIV antigens) [70]		

Abbreviation: EIA: Enzyme Immunoassay; PCR: Polymerase Chain Reaction.

**Table 2:** Examples of testing algorithms for recent HIV infection (criteria for recency) that have been used in various settings.

RITA	Recency Test 1	Recency Test 2	Viral Load (Copies/mL)	ART	Another Marker/Condition (Cells/mm for CD4 T-Cell Count)	Reference
A	LAg Avidity-EIA (<1.5 ODn)	NA	>1000	NA	NA	[56,66,95]
B	LAg Avidity-EIA (<1.5 ODn)	NA	>1000	NA	NAT for HIV seronegative people	[132]
C	LAg Avidity-EIA (<1.5 ODn)	NA	>1000	No ART	NA	[107,131]
D	LAg Avidity-EIA (<1.5 ODn)	NA	>1000	No ART	CD4 T-cell > 200 and no AIDS	[129]
E	Avidity test (<80%)	BED-EIA (<1 ODn)	>400	NA	CD4 T-cell > 200	[130]
F	LAg Avidity-EIA (<2.8 ODn)	BioRad-avidity (<95%)	>400	NA	NA	[127,128]

Abbreviations: AIDS: Acquired Immune Deficiency Syndrome; ART: Antiretroviral treatment (based on drug testing, clinical records or self-report); BED-EIA: An assay whose name originates from the trimeric branched peptide that it uses—from the immunodominant region of the gp41 of HIV-1 subtype B, a Circulated Recombinant Form (CRF_01 AE), and subtype D; EIA: Enzyme Immunoassay; LAg Avidity-EIA: Limiting Antigen Avidity assay; NA: Not applicable; NAT: Nucleic Acid Amplification Test; RITA: Recent Infection Test Algorithm; ODn: Normalized Optical Density.

## Data Availability

Not applicable.
